# Molecular basis of mitogen-activated protein kinase ERK2 activation by its upstream kinase MEK1

**DOI:** 10.64898/2026.01.19.700303

**Published:** 2026-01-19

**Authors:** Jill von Velsen, Pauline Juyoux, Nicola Piasentin, Hayden Fisher, Karine Lapouge, Oscar Vadas, Francesco Luigi Gervasio, Matthew W. Bowler

**Affiliations:** 1 European Molecular Biology Laboratory (EMBL), Grenoble, France.; 2 Université Grenoble Alpes, CNRS, Commissariat à l’Energie Atomique et aux Energies Alternatives, EMBL, ISBG, Grenoble, France.; 3 Institute of Pharmaceutical Sciences of Western Switzerland, University of Geneva, Geneva, Switzerland; 4 School of Pharmaceutical Sciences, University of Geneva, Geneva, Switzerland.; 5 Swiss Bioinformatics Institute, University of Geneva, Geneva, Switzerland; 6 European Synchrotron Radiation Facility, Grenoble, France; 7 Protein Expression and Purification Core Facility, EMBL, Heidelberg, Germany.; 8 Protein Biochemistry Platform, Faculty of Medicine, University of Geneva, Geneva, Switzerland; 9 Department of Chemistry, University College London, London, WC1E 6BT, United Kingdom

## Abstract

The RAS-RAF-MEK-ERK mitogen-activated protein kinase (MAPK) pathway relays extracellular signals into a cellular response and its dysregulation leads to many pathologies, particularly cancer. Here, we determined cryo-EM structures of the MAP2K MEK1 activating its substrate MAPK ERK2, the final event in the cascade. We define the molecular details of specificity and phosphoryl transfer to the tyrosine of the ERK2 activation loop and examine the mechanism of substrate recognition using solution techniques and molecular dynamics. Binding of the substrate MAPK leads to release of the MAP2K catalytic machinery, explaining the mechanism of many disease-causing mutations, and ERK2 release is not required for nucleotide exchange, suggesting a processive mechanism. Our data advance the understanding of MAPK signalling and provide a starting point for drug development.

## One-Sentence Summary

Cryo-EM structures of the MEK1-ERK2 complex reveal details of cellular signal transmission.

Cells need to respond to their environment in order to adapt and react (1). Numerous signalling cascades have evolved in eukaryotes to transmit signals or external conditions, from the cell surface into the cell, thereby eliciting a response (2). The mitogen-activated protein kinases (MAPKs) are a family of highly conserved kinases (3) responsible for cellular reactions elicited by extracellular signals from stress, mitogens, cytokines or growth factors and include the extracellular signal-regulated kinases (ERK1 and 2), the final serine/threonine kinases in the RAS-RAF-MEK-ERK branch of the pathways (4, 5). The ERKs have a wide range of substrates that they can activate, including transcription factors and protein kinases, leading to responses such as cell growth, differentiation, and proliferation. The key role of the pathway in regulating cell behavior leads to its dysfunction being implicated in many diseases, with around 30% of cancers carrying mutations in components of the pathway (6, 7).

The cascades involve multiple kinases, allowing signal amplification as well as positive and negative feedback loops (8, 9). Signals are propagated through the pathways via sequential phosphorylation of activation loops (A-loops) that leads to structural rearrangements, moving kinases from inactive to active states (10). In the case of the MAP kinases, a TxY motif on the A-loop is doubly phosphorylated by their upstream kinases, the MAP2Ks or MEKs, unusual dual-specificity kinases able to phosphorylate both threonine and tyrosine residues. While the MAPKs are promiscuous, the MAP2Ks are highly selective, with ERK1 and 2 being the only known substrates of MEK1 (11). Specificity begins via a kinase interaction motif (KIM or D-motif), located in the disordered N-terminal region of the MAP2Ks, that docks to an allosteric common docking (CD) site on the MAPK (12). The KIM ensures some specificity (13) and prepares the MAPK A-loop for phosphorylation (14), while the predominantly disordered N-terminal linker region, located between the KIM and core kinase domain, guides the MAP2K to the correct docking sites, aligning the kinases for catalysis (15). Once activated, the MAPK moves to cellular compartments and the nucleus, where cell-specific responses are induced via the activation of further protein kinases and transcription factors (5).

There is a wealth of data on the individual components of these pathways (16–20); however, until recently, details of the binding interactions and mechanisms of phosphorylation have been lacking. Cryogenic electron microscopy (cryo-EM) is now revealing many of the interactions between components of the pathways, including scaffolds and modulators, leading to a better understanding of signal transmission (21–24). Our work on the MKK6-p38α branch of the pathway (15) provided the first glimpse of a MAPK (p38α) being activated by its MAP2K (MKK6). It demonstrated how specificity could be maintained between structurally similar signalling components, and showed that substrate recognition was distal from the A-loop, a feature recently also observed in the CDK-CAK complex (25). However, the limited resolution did not allow a full description of activation. Here, we have determined cryo-EM structures of the MAP2K MEK1 (MAP2K1) in complex with its substrate MAPK ERK2 (MAPK1) in inactive, active and nucleotide free states, to overall resolutions between 2.99 Å and 3.63 Å. Integrating these data with X-ray crystallography, hydrogen-deuterium exchange mass spectrometry (HDX-MS), molecular dynamics (MD) simulations and small-angle X-ray scattering (SAXS), we demonstrate that the interaction between the kinases is transient, with a high dissociation constant, but that multiple sites of interaction drive specificity and catalysis. Substrate recognition leads to destabilisation of the regulatory A-helix on the MAP2K linker, a hotspot for disease mutations (26, 27), allowing MEK1 to move to an active conformation, explaining the mechanism of gain of function in some cancers. Comparison with the MKK6-p38α complex allows the structural basis of MAPK specificity to be described. Our data reveal the molecular details of MAP kinase signalling and pave the way for potential new drug development in cancer therapy.

## Engineering a stable MEK1-ERK2 complex

**Fig. 1.**
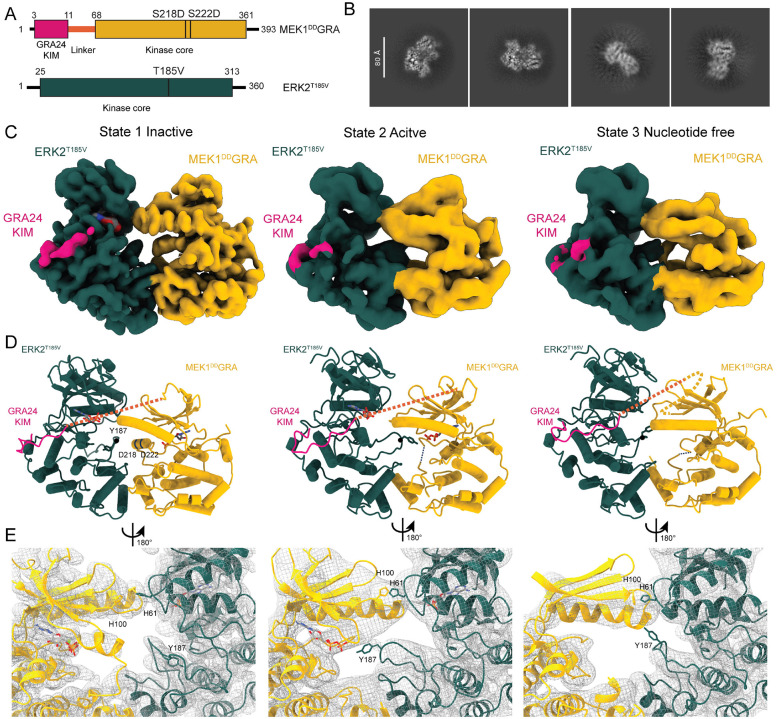
Structures of the MEK1^DD^GRA-ERK2^T185V^ complex. **A** Schematic representation of protein constructs. Numbers indicate domain limits and locations of mutants following Uniprot numbering. Kinase core domain for MEK1 highlighted in yellow, ERK2 kinase core domain shown in dark green. The introduced GRA24 KIM-motif on MEK1 is illustrated in pink, followed by the linker region (residues 12 to 67), represented with an orange line. **B** Representative 2D class averages from the dataset collected on HexAuFoil grids. **C** Cryo-EM reconstructions of MEK1^DD^GRA-ERK2^T185V^ complexes coloured according to A (state 1: inactive; state 2: active; state 3: nucleotide free). **D** Models of MEK1^DD^GRA-ERK2^T185V^ structures, with missing residues indicated by a dashed line. Side chains are displayed on residues of interest and labeled: The A-loop residue Tyr187 on ERK2 is the phosphate acceptor. Asp218 and Asp222 mutant sites render MEK1^DD^GRA constitutively active. **E** The LocScale sharpened Coulomb potential maps (grey mesh) showing the differences in the interface regions between the kinases with models. His100 on MEK1 and His61 on ERK2^T185V^ were identified as a novel site of contact.

The interactions between MAPK pathway components must be transient in order to maintain signal transmission, posing a challenge for structural studies. We employed the same strategy that we applied to the MKK6-p38α complex by using the KIM sequence from the *Toxoplasma gondii* effector protein GRA24 (28, 29) to stabilise the interaction between MEK1 and ERK2. We found that the GRA24 KIM also binds ERK1 and 2 with high affinity (K_D_ = 0.31 μM for both), at least 100-fold higher than the MEK1 KIM peptide (Fig. S1A). To obtain high-resolution information on the binding mode, we determined the crystal structure of human ERK1 (MAPK3) bound to a peptide corresponding to the sequence of the GRA24KIM1 at 2.17 Å resolution (Fig. S1 and Table S1). Human ERK1 was used as we were unable to obtain crystals with ERK2 (88% similarity between the two proteins). The binding of the GRA24KIM1 induces the same conformational change as the binding of the KIM sequence from MEK2 on ERK2 (4h3q (30), RMSD between the Cα of the kinases 0.79 Å, Fig. S1C). Further, we introduced phosphomimetic mutations in the MEK1 A-loop (S218D and S222D) to produce a constitutively active MEK1 chimera with the GRA24 KIM, named MEK1^DD^GRA (Fig. 1A). The addition of the GRA24 KIM increases residue numbering by 6; for clarity, we use the wild-type MEK1 numbering (uniprot ID Q02750) throughout the manuscript. To limit complex heterogeneity through two possible phosphorylation events, we further stabilised the complex by mutating ERK2 to have only the A-loop tyrosine available for phosphorylation (ERK2^T185V^, Figs. 1A, S2 and 3). Finally, we stabilised the complex with ADP and the transition state analogue aluminium tetrafluoride (AlF_4_^−^) (31). We were also able to purify a complex with the native KIM, MEK1^DD^-ERK2^T185V^ (Fig. S2). However, structural studies led to poor 2D class averages and we pursued the MEK1^DD^GRA-ERK2^T185V^ complex for cryo-EM (Fig. S2B).

We imaged the 86 kDa MEK1^DD^GRA-ERK2^T185V^ complex using cryo-EM. Initial micrographs recorded on UltraAufoil grids showed promising results and reasonable class averages, but use of HexAufoil grids (32) yielded improved two-dimensional (2D) class averages with clear secondary-structure features and low background (Fig. 1B), allowing the reconstruction of three different states of the complex from a single data set of 88,517 movies. Multiple rounds of picking and training using Topaz (33) were necessary to generate a stack of good-quality particles. A combination of 3D classification and heterogeneous *ab initio* reconstruction (34) including high-resolution information (HR-HAIR (35)) improved particle alignments and allowed the separation of three distinct states (Fig. 1 and S4 and S5). The most abundant state, resolved to an average resolution of 2.99 Å, is a non-productive complex where the A-loop of MEK1 is in the inactive conformation, and the A-loop of ERK2 cannot access the active site. In the second state, resolved to an average resolution of 3.47 Å, the A-loop of MEK1 is disordered, and the A-loop of ERK2 extends into the active site of MEK1 with the tyrosine 187 (of the TxY motif) approaching the catalytic aspartate (Asp190) and nucleotide. In the final state, resolved to an average resolution of 3.63 Å, the kinases are still complexed together, but no nucleotide is bound, and the A-loop of ERK2 retracts from the active site with the tyrosine facing away (Fig. 1D and E, and S6).

## Inactive MEK1 can bind its substrate with face-to-face architecture at multiple low-affinity sites

**Fig. 2.**
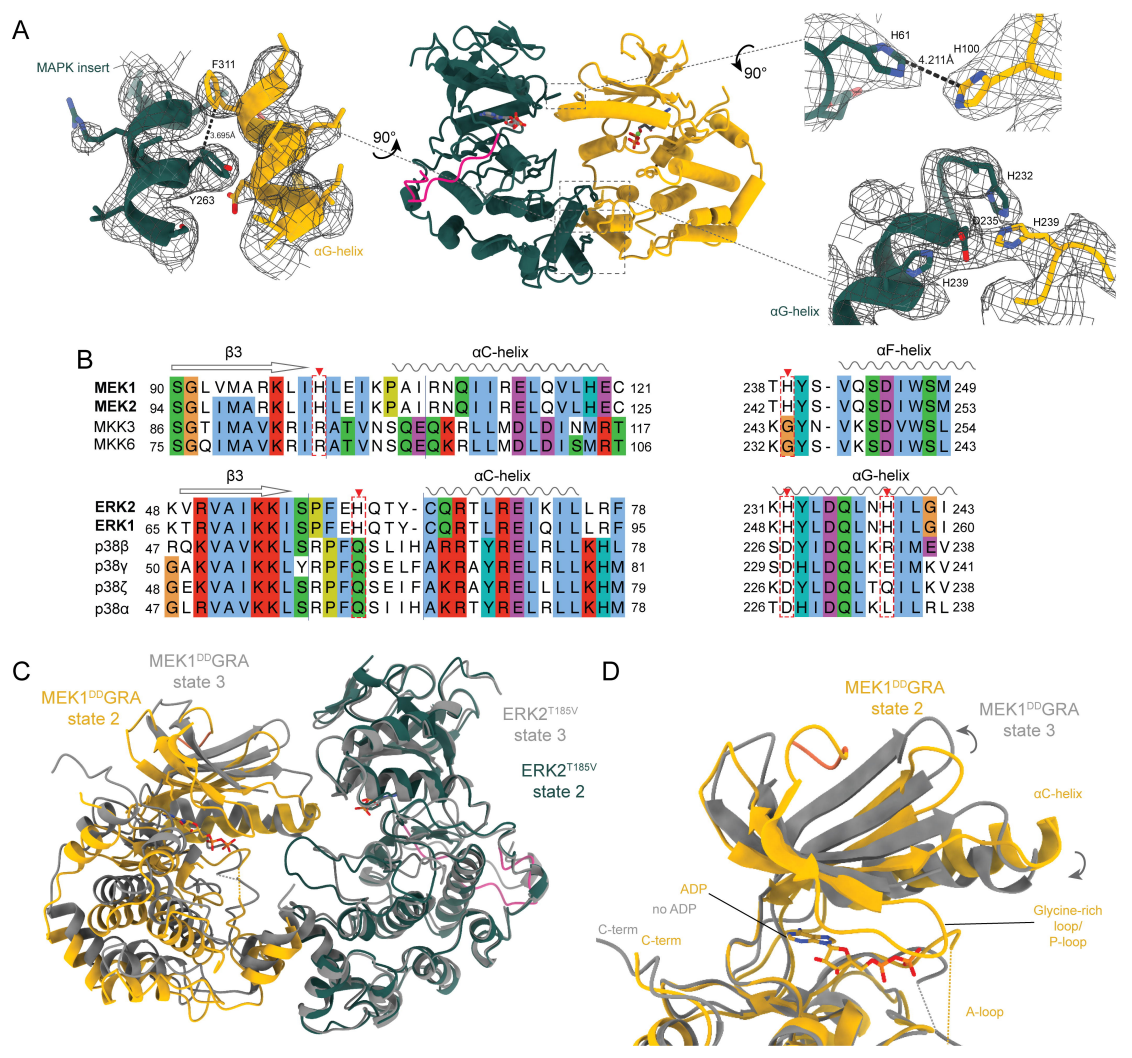
Interaction sites in the MEK1^DD^GRA-ERK2^T185V^ complex. **A** Beyond the KIM, MEK1 interacts with ERK2 at the hydrophobic pocket formed by the MAPK specific insert via the F-motif, a histidine triad coordinating a glutamate between the αG-helix of ERK2 and the loop between the αF-helix and MEK1 A-loop, and a permissive His-His interaction in the N-lobes. Sharpened Coulomb potential from the State 1 reconstruction is displayed as a grey mesh. **B** The histidines are conserved in the MEK-ERK branch but not the other MAPK pathways and are conserved across metazoans (Fig S9)**. C** State 2 and 3 aligned on the substrate ERK2. Nucleotide free MEK1 is rotated 5.6° and shifted up by 2 Å to slightly detach from its substrate. **D** The N-lobe of state 3 has opened 12.5° relative to the C-lobe of active MEK1, releasing nucleotide.

As the inactive complex had the highest overall resolution, with the maps showing excellent details of side chains, we used this state to build the initial model and compare it to the subsequent states (Fig. S5 and 6). The kinase domains are clearly resolved, in similar positions to the AlphaFold3 (AF3) (34) prediction, that also predicts the inactive conformation of the MEK1 A-loop (Fig S7). Two features were surprising. First, the N-terminal linker, following the KIM motif, is completely disordered and not visible, despite two confidently predicted helices (Fig. S7) one of which, the A-helix, is often resolved in crystal structures of MEK1 and is thought to have a regulatory role by stabilising the inactive conformation (36). Second, the A-loop of MEK1 is in the inactive conformation, despite phosphomimetic mutations. In solution, the A-loop is probably in equilibrium between active and inactive states, with phosphorylation driving it to the active side (23). The aspartate mutations appear not to be as effective as phosphorylation, biasing the equilibrium enough for enzymatic activity but not completely to the structurally active state. In crystal structures of MAP2Ks with the same mutations, the A-loop is not visible (18), implying high flexibility. The equilibrium between conformations would also explain why the wild type MAP2Ks exhibit basal activity (37), as it will occasionally bind its substrate MAPK in an active conformation, in a similar manner to our state 1 structure. The highly disordered MEK-specific proline-rich loop is also not visible. In this inactive, non-productive state, a short helix in the A-loop of MEK1 blocks the entry of the A-loop of ERK2 from approaching the nucleotide. The overall interactions are similar to the MKK6-p38α complex (15): the KIM interacts at the CD-site in a similar manner to the isolated KIM peptide (Fig. S8), and the αG-helix of MEK1 binds through mainly hydrophobic interactions, led by the phenylalanine of the F-motif, at the MAPK-specific insert of ERK2. In the MEK1-ERK2 complex, there are additional interactions: two histidine residues on the N-terminal lobes of the kinases and a triad of histidines coordinating an asparagine, one histidine from the loop between the MEK1 αF-helix and A-loop, and two histidines and an asparagine on the αG-helix of ERK2 (Fig. 2A). These histidines are conserved in MEK1 and 2 and ERK1 and 2 but are absent in the other branches of the MAPK cascades (Fig. 2B and S9). Histidines are advantageous for transient interactions as they are geometrically permissive and slightly weaker than classical salt bridges (38, 39) as well as being able to respond to pH differences (40). These sites are an additional check on substrate recognition, specific to the MEK-ERK pathway.

## The active state binds its substrate at multiple sites distal from the A-loop

A second state of the complex showed clear density for the A-loop of ERK2 entering the active site of MEK1 and the A-loop of MEK1 is disordered. This state was determined at an overall resolution of 3.47 Å, but the main features of the kinases are well resolved. The relative orientation of the two kinases is the same as the inactive complex, maintaining the same sites of contact, including the MEK-ERK-specific histidine interactions, despite the movement of the αC-helix. The catalytic αC-helix has moved towards the “in” conformation, and the A-loop of ERK2 extends towards the active site of MEK1. The density for the αC-helix is blurred, implying movement (Fig 1C and S6D), and there is no clear position for Lys97, making it difficult to see if the catalytic spine is in place. We were unable to further separate the class of particles with a better-defined position. A model derived from the active conformation, including the salt bridge, is not inconsistent with our maps (Fig. S6), and the helix is likely in a continuum of positions. However, we chose to model an intermediate location to be consistent with the data, state 2, which we call ‘active’, and produced another model, used exclusively for MD simulations, that we call the ‘fully active’ conformation that has the salt bridge in place. Although the complex was supplemented with ADP.AlF_4_^−^, we do not observe the metal fluoride, possibly due to the dynamic nature of the interaction. In our model, the tyrosine is 6.2 Å from the MEK1 catalytic aspartate 190 and 4.7 Å from the $\text{AD}\underset{˙}{\text{P}}$β-phosphate. As with the MKK6-p38α complex, no contact between the MAPK A-loop and the MAP2K is observed, further supporting our hypothesis that substrate recognition occurs at sites remote from the active site and the A-loop is relatively free to move within the confines of the MAP2K active site. This allows either the tyrosine or threonine to access the active site for nucleophilic attack of the γ-phosphate.

## The nucleotide free state suggests a route for a processive mechanism

The third state, where nucleotide is absent from MEK1, was determined at an average resolution of 3.63 Å. The same contact sites are maintained as in the other states; however, MEK1 has rotated 5° relative to ERK2, maintaining the N-lobe connection, but the αG-helix moves away from the MAPK insert (Fig. 2C). The most significant difference is the absence of nucleotide, along with complete disorder of the P-loop (or glycine-rich loop) and the retraction of the ERK2 A-loop (Fig. 2D). The P-loop has an important role in coordinating phosphates of ATP in the active site and positioning them for catalysis. In this state, the loop itself, and most of the β1-β2 strands, are disordered, and the nucleotide binding site is empty. However, the histidine interactions observed in the previous states are maintained (Figs. 1 and 2). The MEK1 K_d_ for ADP is 2 μM (41), well below the buffer condition of 250 μM, implying that this conformation has significantly reduced affinity for nucleotide. It has long been debated whether phosphorylation of the MAPKs by their MAP2Ks occurs via a distributive mechanism, where the MAP2K dissociates from the MAPK between phosphorylation events, or a processive mechanism, where nucleotide is released and rebound to allow the second phosphorylation, without dissociation of the kinases. There is strong experimental evidence for both mechanisms, with a dissociative mechanism being more likely *in vitro* (37, 42, 43) and a processive mechanism becoming more likely in crowded conditions or in the cell (44). This structure demonstrates that a MAP2K can bind its substrate MAPK in a similar manner to the active state, and exchange nucleotides, demonstrating how a processive mechanism could work.

## A complex network of sites primes the kinases for catalysis and ensures specificity

**Fig. 3.**
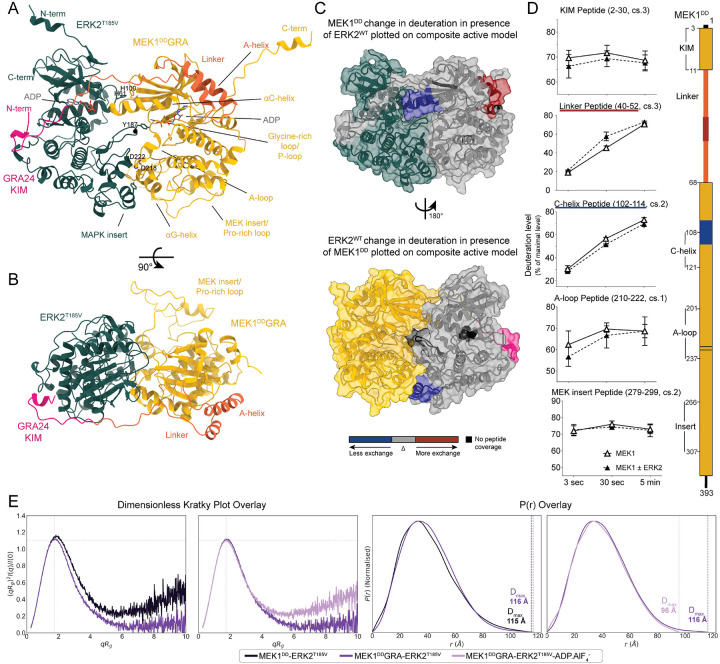
Dynamics of the MEK1-ERK2 complex. **A** Composite model of the MEK1^DD^GRA-ERK2^T185V^ complex (active, state 2) constructed from the cryo-EM model and the AF3 prediction to complete disordered regions (full prediction in Fig. S7). **B** Top view of the same model. **C** Interaction sites identified with HDX-MS on MEK1^DD^ (top) and ERK2^WT^ (bottom) plotted on the composite active model. Binding partners colored as previously are shown for orientation. Regions showing protection upon the addition of ERK2^WT^ or MEK1^DD^, indicative of interaction, are highlighted in blue. Regions showing increased exposure upon binding, indicative of allosteric conformational change, are highlighted in red. **D** Plots for relevant peptide regions of MEK1^DD^, showing deuteration uptake over time for MEK1^DD^ alone or in the presence of a 1.5 fold excess ERK2^WT^. Schematic of MEK1 sequence shown next to the plots for clarity. **E** From left to right: Dimensionless Kratky plot from SEC-SAXS experiments for MEK1^DD^-ERK2^T185V^(dark purple) and MEK1^DD^GRA-ERK2^T185V^(purple) complexes overlaid for comparison, the introduction of the GRA KIM yields a more globular particle with reduced flexibility. The second dimensionless Kratky plot shows overlay of MEK1^DD^GRA-ERK2^T185V^(purple) and MEK1^DD^GRA-ERK2^T185V^-ADP.AlF_4_^−^(light purple); the addition of nucleotide and transition state analogue further compacts the complex but increases local flexibility. The third panel shows P(r) distribution for MEK1^DD^-ERK2^T185V^(dark purple) and MEK1^DD^GRA-ERK2^T185V^(purple) which have similar d_max_ values. In the fourth panel, the P(r) distribution for MEK1^DD^GRA-ERK2^T185V^(purple) and MEK1^DD^GRA-ERK2^T185V^-ADP.AlF_4_^−^(light purple) are seen, where a lower D_max_ for sample containing ADPAlF_4_^−^ addition indicates complex compaction.

HDX-MS analysis of the MEK1^DD^-ERK2^WT^ complex was performed to support the observations from cryo-EM (Fig. 3, S2 and S10) (45). Several regions of either MEK1^DD^ or ERK2^WT^ were protected from hydrogen-deuterium exchange when in the complex, correlating with the cryo-EM models of the MEK1^DD^GRA-ERK2^T185V^ complex, such as the MAPK insert of ERK2, where the αG-helix of MEK1 binds, and the αC-helix and the β3-αC connecting loop of MEK1 - including H100 that interacts with ERK2 H61 (Fig. 2A and Fig3C). This confirms that the interactions observed in our stabilised complex are the same in the wild type. In addition to the areas showing protection, the A-helix in the N-terminal linker of MEK1 showed significant deprotection upon binding of ERK2, suggesting an unfolding of the helix (Fig. 3C). This helix has been assigned a regulatory or inhibitory role, and is thought to stabilise the αC-helix in the inactive “out” position. This implies that the binding of the KIM motif in the N-terminus of MEK1 leads to changes allowing greater conformational freedom, ultimately affecting activity. The A-helix is a hotspot for mutations found in cancer and other diseases. Interestingly, these mutations have been found to destabilise the A-helix, leading to partial unfolding, as well as higher P-loop flexibility (46, 47).

The proline-rich loop, specific to MEK1 and 2, shows high flexibility (Fig. 3A and E). The insert has been implicated in binding to scaffolds and upstream components of the pathway, and is essential for ERK activation (48). We observed residual density in the three states of the MEK1^DD^GRA-ERK2^T185V^ complex near the ERK2 A-loop (Fig. S11). The density is within reach of the proline-rich loop; however, it is not sufficiently clear to place any residues. A threonine residue in the loop (T292) is also a site of negative feedback phosphorylation (49, 50) which could prevent the interaction with ERK. This area is therefore a potential additional region of interaction between the kinases, but like the other sites, it is low-affinity and transient, contributing to overall complex formation and specificity, even though no protection was observed in HDX-MS in this region. It should be noted that the KIM sequence of MEK1 does not show increased protection upon addition of ERK2 - highlighting the highly dynamic nature of the interactions, as this is a well-characterised and defined binding site. Upon inspection of the uptake plots for the KIM peptides, a trend of protection can be observed (Fig. 3D), but remains not significant in triplicate, likely also due to the very fast exchange rate already at 3s.

The observation of A-helix unfolding, combined with our structural data, suggests a mechanism for the gain-of-function of mutations in this region, whereby they are primed for activity before substrate engagement, and may have increased flexibility once bound, leading to higher activity, or indeed increased activity in the absence of A-loop activation by phosphorylation, leading to aberrant signalling.

## Solution studies of the MEK1^DD^GRA-ERK2^T185V^ and MEK1^DD^-ERK2^WT^ complexes show a highly dynamic interchange between the kinases

We used isothermal titration calorimetry (ITC) to measure the interaction between the MEK1^DD^GRA-ERK2^WT^, MEK1^DD^-ERK2^WT^ and MEK1^WT^-ERK2^WT^ kinases. While we were able to determine dissociation constants for MEK1^DD^GRA and ERK2^WT^ (1.94 μM) we were unable to measure dissociation constants between either MEK1^DD^ and ERK2^WT^ or MEK1^WT^ and ERK2^WT^ (Fig. S12 and Table S3), implying that the K_D_s are higher than 50 μM, consistent with the value of 29 μM using fluorescent labeled proteins in cells (51). The concentration of MEK and ERK in the cell is estimated at 1 μM, implying that with a K_D_ in the 50 μM range, around 1–2% of the proteins will be bound together at any time. This is compatible with the requirement of the kinases to transmit signals via phosphorylation, but not monopolise either the MAPK or MAP2K, so they are free to continue to activate targets. Despite this low affinity, we were able to purify the MEK1^DD^-ERK2^WT^ and MEK1^DD^-ERK2^T185V^ complexes at a starting concentration of 50 μM, where they eluted in a single peak (Fig. S2) during SEC. This is in contrast to our studies of the MKK6-p38α complex, where we were unable to purify the native complex. The ability to purify the native complex opened the possibility to study the complexes by SAXS.

We studied both the MEK1^DD^GRA-ERK2^T185V^ and MEK1^DD^-ERK2^T185V^ complexes by size-exclusion chromatography-coupled small-angle X-ray scattering (SEC-SAXS) in the absence of nucleotide and in the ADP.AlF_4_^−^ supplemented state (Fig. S13 to S15 and Table S4). Analysis of the elution traces revealed conformational heterogeneity in the non-stabilised complex (MEK1^DD^-ERK2^T185V^). While the molecular weight (MW) estimates across the peak remain relatively consistent with a 1:1 heterodimer, the Radius of Gyration (R_g_) exhibited a continuous decrease (34 Å to 28 Å), indicative of a polydisperse ensemble of extended and compact conformations resolving on the column. Introduction of the GRA-KIM (MEK1^DD^GRAERK2^T185V^) stabilises the complex: the R_g_ across the elution peak is stable (c. 32 Å), indicative of a monodisperse sample with uniform shape. This was further supported by the dimensionless Kratky plot, which showed a shift in peak position and curve shape reflective of a more compact particle for MEK1^DD^GRA-ERK2^T185V^ when compared to MEK1^DD^-ERK2^T185V^ (Fig. 3E and S14).

To assess the agreement between the cryo-EM structure and the conformation in solution, we generated theoretical scattering profiles from the composite model of the inactive state using PEPSI-SAXS. This yielded reasonable goodness-of-fit values (χ^2^) to the experimental data of 2.70 for MEK1^DD^GRA-ERK2^T185V^ and 3.61 for MEK1^DD^GRA-ERK2^T185V^-ADP.AlF_4_^−^ after refinement of the model using non-linear normal mode analysis. The addition of ADP.AlF_4_^−^ to the MEK1^DD^GRA-ERK2^T185V^ complex induced a global compaction of the core, but an increase in local flexibility as well. The dimensionless Kratky plot of the ligand-bound species showed a characteristic upward deviation at higher qR_g_ values (qR_g_ > 6) compared to the apo form, reflective of increased local flexibility, while the pair distance distribution function, *P*(r), shows a smaller D_max_ for the ligand-bound form (Fig. 3E). Collectively, this suggests that in presence of ADP.AlF_4_^−^ the complex locks into a compact architecture, while simultaneously inducing local plasticity or elongation of linker or loop segments, as observed by HDX-MS (Fig. 3C). This highlights the heterogeneity present in the active site, as observed in the cryo-EM dataset, particularly in the active conformation, which enabled the determination of three states from a single dataset.

## Molecular dynamics simulations show that competent catalytic states can be formed in the active state and highlight the dynamic nature of the interaction

**Fig 4.**
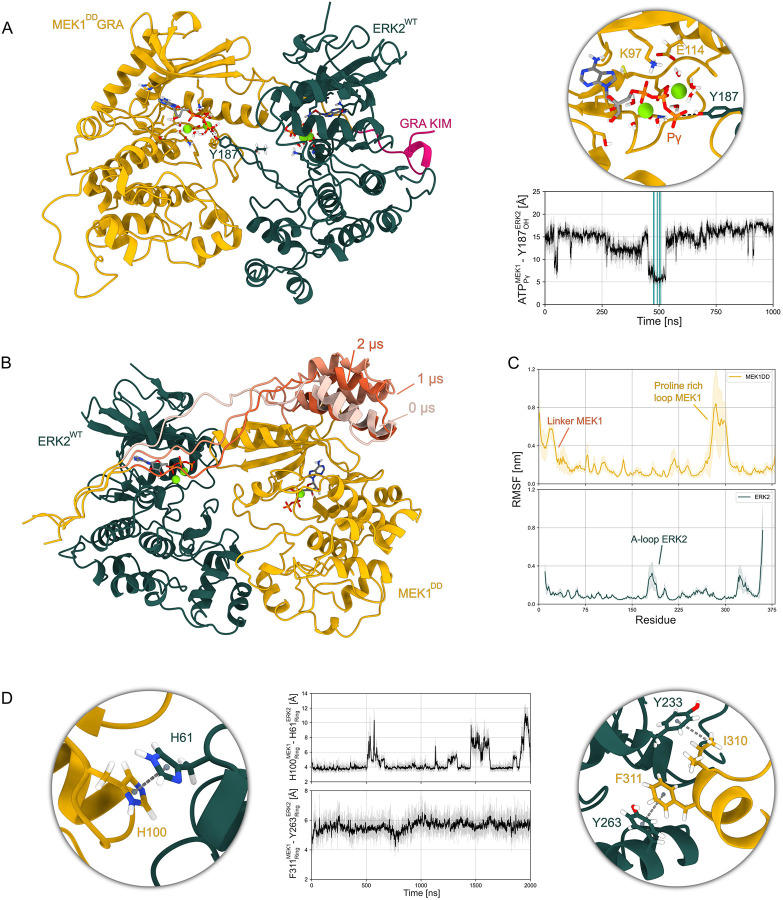
MD simulations show that ERK2 Y187 can reach MEK1 ATP and that the flexibility of the system is consistent with HDX-MS and cryo-EM data. **A** Frame extracted from an MD simulation of the fully active state in which Y187 of ERK2^WT^ approaches the γ-phosphate of MEK1^DD^GRA ATP at a catalytically compatible distance (~3.8 Å). The inset shows K97 interacting with the ATP α-phosphate as well as with E114 to form the salt bridge that stabilizes the αC-in active state. In the distances over time plot, the frames where ERK2^WT^ Y187 reaches MEK1^DD^GRA ATP at a catalytically compatible distance are highlighted in blue. **B** Three frames extracted from an MD simulation of the inactive state of the MEK1^DD^-ERK2^WT^ at different times where the α-helices of the N-terminal linker of MEK1^DD^ show large rearrangements. **C** Average root mean square fluctuation (RMSF) for all the MEK1^DD^GRA-ERK2^WT^ MD simulations (24 replicas, 48 μs). **D** Distances between MEK1^DD^GRA H100 and ERK2^WT^ H61 at the N-lobe interface and between the MEK1^DD^GRA F311 and ERK2^WT^ Y263 at the MAPK-specific insert αG-helix interface. The insets show frames extracted from the corresponding time series.

To investigate the dynamics of the complex, and integrate the observations collected from our structural and solution data, we also performed MD simulations starting from a set of 48 replicas, each of 2 μs (Table S5). The simulations started from the state 1 and 2 models obtained from the cryo-EM maps: First we reverted the A-loop of ERK2 to the wild type and combined the model with AF3 predictions to produce a composite model (Fig. 3A and B) of MEK1^DD^GRA-ERK2^WT^ (Fig. 4A). We further reverted the KIM of MEK1 to wild type to produce a composite model of the MEK1^DD^-ERK2^WT^ complex (Fig. 4B). Additionally, we produced a model with the catalytic salt bridge formed, that is consistent with the maps of state 2 (Fig. S6D). The simulations explored several states of the active and inactive conformational ensembles. The synthetic HDX-MS profiles computed from these simulations show reasonable agreement with the experimental ones (Fig. S16), suggesting that the ensembles explored capture key features of the dynamic behavior observed in solution. Occasionally, the simulations explored catalytically competent states in which Y187 of ERK2 approached the γ-phosphate of the ATP in the active site of MEK1 at distances compatible with phosphate transfer (Fig. 4A). Interestingly, in no simulations did T185, the other phosphorylation site of ERK2, approach the γ-phosphate of ATP at the timescales investigated.

The analysis of the flexibility of the complex revealed characteristics consistent with the cryo-EM and HDX-MS data (Fig. 4B, C and S16 and 17). In ERK2, the most flexible domain is the A-loop, even in the inactive state (Fig. S18), demonstrating that in the crowded environment the kinase still retains partial freedom of movement. In MEK1 the most flexible domains are the linker and the proline-rich loop, the latter exhibiting considerable conformational freedom. Remarkably, in some simulations the A-helix of the N-terminal linker of MEK1 detached and showed increased flexibility (Fig. 4C), consistent with both the absence of this domain in the cryo-EM maps and its increased deuterium exposure in the HDX-MS measurements.

In all of the simulations, the kinases stayed at least partially in contact and did not fully dissociate. As for the MKK6-p38α complex, the C-lobe proved to be the critical interface for the dimer stability (Fig. 4D and S19). The N-lobe interface, however, is much more ephemeral, and can be lost and re-formed while the complex is held together by the C-lobe interface and the linker (Fig. S20). In a number of simulations MEK1 H100 and ERK2 H61 were close at the N-lobe interface (Fig. 4D), supporting the experimental findings that this interaction contributes to substrate specificity but is not so tight as to prevent substrate release.

Overall, the two coordinating magnesium ions in each of the binding sites of the active complexes show high mobility (Fig. S21). While the ions do not leave the binding site and remain coordinated to the ATP phosphates, in most of the simulations at least one of the two Mg atoms leaves the proximal oxygen of the aspartate of the DFG motif. This mobility is in line with the difficulty to resolve the ion positions in the maps, and might suggest that the second coordinating metal ion is only transiently present in the binding site at the moment of phosphorylation. Conversely, the single coordinating ion in all the inactive simulations (totalling 32 μs) is extremely stable (Fig. S21). In this configuration, the partially folded A-loop of MEK1 limits the available space for the aspartate of the DFG domain, hinting that the inactive loop might hinder down-stream phosphorylation both by obstructing the access to the binding site and by limiting the aspartate freedom to host a second ion at the binding site. These simulations confirm the binding sites observed in the cryo-EM structures and highlight the overall flexibility of the complex, particularly the active site of MEK1, that is in constant turnover even in the active, substrate bound form.

## Discussion

We have determined snapshots of various stages of the activation of the MAPK ERK2 by its upstream MAP2K MEK1. Combining these structures with biophysical studies in solution, as well as molecular dynamics simulations, we provide insight into the mechanisms of substrate recognition and catalysis in this important signalling pathway. Our study has revealed a complex network of interactions between the MAP2K and its substrate MAPK. The KIM is responsible for initial docking and preparation of the MAPK for phosphorylation by exposing the A-loop. However, it also transmits instructions to the MAP2K via its linker. We previously demonstrated that the linker between the KIM and kinase core is an important feature of specificity (15). We hypothesised that the length was important in guiding the αG-helix to the MAPK insert. In MEK1, the initially helical linker plays an additional role in recognising substrate binding and preparing MEK1 for activity by freeing the αC-helix and increasing flexibility in the P-loop, thereby facilitating nucleotide exchange. Once the KIM and αG-helix are engaged, the ERK2αG-MEK1αF triple histidines, and the N-lobe interaction via MEK1-His100 and ERK2-His60, position the two kinases correctly for catalysis. It is also possible that at this point the proline-rich loop - specific to MEK1 and 2 - engages with the ERK2 A-loop as an additional interaction. At this stage, even when all the specific interactions are fulfilled, if the MEK1 A-loop remains in the inactive conformation, it would not be able to activate ERK2, as the short helix blocks access to the nucleotide. If the MEK1 A-loop is in the active conformation, the A-loop of ERK2 can approach the catalytic site of MEK1 with a large amount of freedom. Our structure of the active conformation (state 2) shows that there is no interaction between the MAPK A-loop and the MAP2K beyond the nucleophilic oxygen atom approaching the ATP. Our solution and MD simulation data demonstrate considerable dynamics in the active site and loops of the complex when bound to each other, implying that the site is in constant turnover between aligned and unaligned active states (Figs. 4, S18 and 20). This would allow the first phosphoryl transfer to occur while avoiding any low-energy wells to stall the second step. Our structure of the nucleotide free state demonstrates that the MAP2K can efficiently exchange nucleotides, as the P-loop is disordered, allowing release, while being engaged with its substrate. The A-loop of the MAPK is free to retract, and given the conformational freedom, the phosphorylated residue should not hamper the approach of the second residue to the active site, showing how a processive mechanism could proceed. Together, these data demonstrate a complex exchange of information between enzyme and substrate, leading to efficient and highly specific activation, a hallmark of this signalling cascade.

**Fig. 5.**
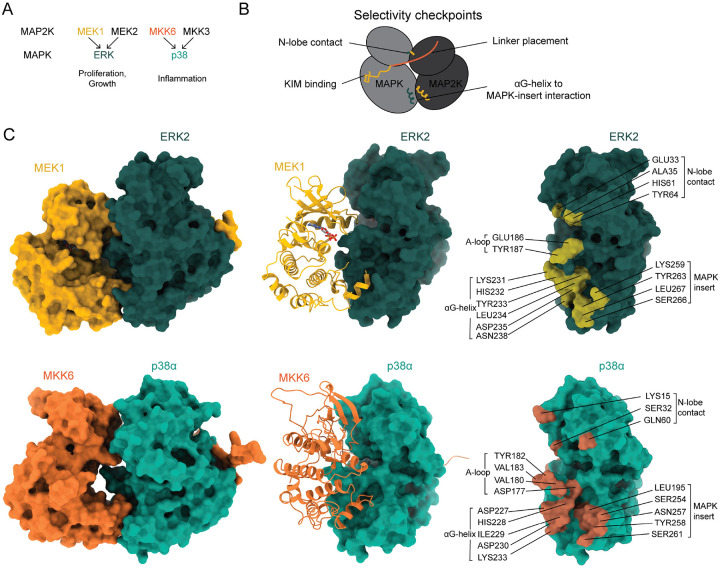
Molecular details of MAPK pathway specificity. **A** Schematic showing the specificity of MAP2Ks for MAPKs and the consequences of signalling, nodes with structural data on the interaction are coloured. **B** Diagram describing the selectivity checkpoints in the MAP2K-MAPK interactions of the MAP kinase pathways. **C** Comparison of the MEK1^DD^GRA-ERK2^T185V^ complex with the MKK6^DD^GRA-p38α^T180V^ complex. While the overall face-to-face conformation is maintained between pathways significant differences exist in the relative positioning of the kinases (a 36° rotation of MKK6 relative to ERK2 leads to a higher N-lobe interaction) and the residues that interact. From left to right the complexes are displayed as space filling surfaces, the substrate MAPK as a surface and the MAP2K as a ribbon, and the surface of the substrate MAPK with the interface residues highlighted.

The determination of a second MAP2K-MAPK complex structure allows the basis of pathway specificity to be described. Comparison of the MKK6-p38α complex with the MEK1-ERK2 complex shows similarities in global interactions, but significant differences that prevent signalling crosstalk. Even though the KIM and αG-helix bind in a very similar manner, and the face-to-face orientation is conserved, the relative position of the enzyme to the substrate is significantly different (Fig. 5). Superposition of the substrate MAPKs (p38α and ERK2) shows that MKK6 is rotated 36°, relative to MEK1, towards the MAPK substrate with a 5.7 Å translation along the axis, such that the MKK6 N-lobe is higher relative to its substrate than MEK1 (Fig. 5C). The rotation axis is centred around the point just after the KIM, at which the N-terminus of the MAP2K departs towards the N-terminal lobe, highlighting the linker length as an important feature for correct substrate engagement (Fig. S22A and B). It is also due to differences in the αG-helix binding to the hydrophobic pocket formed by the MAPK-specific insert (Fig. 5). The MKK6 loop between the αF-helix and A-loop also abuts p38α in a similar manner to MEK1, however the residues are quite different. The moderate resolution of the MKK6-p38α structure did not allow confident residue assignment in this interaction, but residues in this area are also conserved in this branch, indicating another possible site determining specificity (Fig. 5). Once the αG-helix is docked, and the C-lobe histidine triad is in place, the N-lobe interactions must then be fulfilled. The major difference in the interfaces is between the N-lobes of the kinases: a His-His interaction (Fig. 2A), maintained only in the MEK-ERK branch, is the final site that likely determines specificity. If the substrate MAPK is incorrect, the N-lobe interaction will not be stabilised, preventing alignment of the MAPK A-loop with the MAP2K active site. The MEK branch may also have the proline-rich loop as an additional site of interaction at the ERK A-loop, adding a further verification site. It is interesting to note that the interface with MEK1 substrate ERK2 is quite different to its activating kinase CRAF (Fig. S22C and D). In addition to these substrate-specific interactions, the N-terminal linker serves as another check on the substrate by releasing the catalytic machinery only upon correct binding. The p38α branch does not contain helices in the N-terminal linker, but MEK1 and 2, as well as MKK7, do contain helical elements. Swapping the MKK6 linker for those from MEK1 and 2 abolishes p38α signalling in cell assays (15). In addition to linker length, the regulatory helices also play an important role in substrate discrimination.

Our structural data delineate several snapshots in the activation of ERK2 by MEK1. As with the MKK6-p38α complex, substrate recognition is at sites remote from the active site, allowing considerable flexibility in the residues that can be phosphorylated while maintaining high specificity. In combination with structural studies in solution, and molecular dynamics studies, our data point to a complex that is highly dynamic during catalysis, allowing fast turnover, essential in signalling, and avoiding low energy states, with an efficient processive mechanism likely *in vivo* (44). Together, we describe the structural basis of ERK activation, a fundamental step in many cellular processes and a potential checkpoint for cancer and other diseases. While there are several drugs in clinical use that target the MEKs, ERK1 and 2 do not have drugs in use. Our structural data could facilitate the development of new therapeutics.

## Supplementary Material

Materials and Methods

Figs. S1 to S22

Tables S1 to S5

References (*52–106*)

Data S1 and S2

## Data Availability

Accession codes: Structure of ERK1-GRA24KIM1: https://doi.org/10.15151/esrf-dc-2310714581 (raw diffraction data); PDB-9TU0 (coordinates and structure factors; Protein Data Bank). Structures of the MEK1^DD^GRA-ERK2^T185V^ complex: https://doi.esrf.fr/10.15151/ESRF-ES-2012234353 (raw micrographs), EMD-56418 (state 1), EMD-56419 (state 2) and EMD-56420 (state 3) (map; Electron Microscopy Data Bank) and PDB-9TYG (state 1), PDB-9TYH (state 2) and PDB-9TYI (state 3) (coordinates; Protein Data Bank). The SAXS curves and pair distribution functions are deposited in the Small Angle Scattering Biological Data Bank (access codes: SASBDB-SAS7328, SASBDB-SAS7329, SASBDB-SAS7330 and SASBDB-SAS7331).
